# Effect of Lemon Essential Oil Microemulsion on the Cariogenic Virulence Factor of *Streptococcus mutans* via the Glycolytic Pathway

**DOI:** 10.3290/j.ohpd.b3464891

**Published:** 2022-10-19

**Authors:** Shuyu Luo, Chong Feng, Yafei Zheng, Yanwei Sun, Changqing Yan, Xiangyu Zhang

**Affiliations:** a Doctoral Student, School and Hospital of Stomatology, Tianjin Medical University, Tianjin, China. Idea, hypothesis, experimental design and implementation, wrote manuscript.; b Doctoral Student, School and Hospital of Stomatology, Tianjin Medical University, Tianjin, China; Department of Orthodontics, Tianjin Stomatological Hospital, School of Medicine, Nankai University, Tianjin, China. Idea, hypothesis, experimental design and implementation, wrote manuscript.; c Graduate Student, School and Hospital of Stomatology, Tianjin Medical University, Tianjin, China. Data management, wrote manuscript.; d Dentist, Department of Pediatric Dentistry, Tianjin Stomatological Hospital, School of Medicine, Nankai University, Tianjin, China. Experimental design and implementation, data management.; e Graduate Student, School and Hospital of Stomatology, Tianjin Medical University, Tianjin, China. Data management.; f Professor, School and Hospital of Stomatology, Tianjin Medical University, Tianjin, China. Idea, hypothesis, revised manuscript.; * Shuyu Luo and Chong Feng contributed equally to this study.

**Keywords:** dental caries, lemon essential oil microemulsion, *Streptococcus mutans*, acid production, lactate dehydrogenase

## Abstract

**Purpose:**

To investigate the effects and mechanisms of lemon essential oil products on dental caries prevention.

**Materials and Methods:**

Lemon essential oil microemulsions (LEOM) with concentrations of 1/8 minimum inhibitory concentration (MIC), 1/4 MIC, and 1/2 MIC were applied to *S. mutans* at concentrations of 0.2%, 1%, and 5% glucose, respectively. Changes in acid production capacity of *S. mutans* were measured based on changes in pH. The effect of the reductive coenzyme I oxidation method on LDH activity was examined. The effect of lemon essential oil microemulsion on the expression of the lactate dehydrogenase gene (ldh) was detected by a quantitative real-time polymerase chain reaction.

**Results:**

Lemon essential oil microemulsion at 1/2 MIC concentration reduced the environmental pH value at different glucose concentrations, compared to those observed in the control group (p < 0.05). LDH activity of *S. mutans* was decreased at three subinhibitory concentrations of lemon essential oil microemulsions (p < 0.05). The effect of lemon essential oil microemulsions on *S. mutans* LDH activity and bacterial acid production were positively correlated (r = 0.825, p < 0.05). Lemon essential oil microemulsion at 1/2 MIC concentration downregulated the expression of the ldh gene of *S. mutans* at different glucose concentrations (p < 0.05). In different glucose environments, lemon essential oil microemulsions at subminimum inhibitory concentrations can inhibit the acid production of *S. mutans* by reducing ldh expression and LDH activity in the glycolytic pathway, proving its anti-caries potential.

**Conclusions:**

LEOM can effectively prevent dental caries and maintain the microecological balance of the oral environment.

Dental caries is a disease of dental hard tissues and has a multifactorial etiology.^15,18^ In China, the 2017 Fourth National Oral Health Epidemiological Survey revealed that the rate of caries in the deciduous teeth of 5-year-old Chinese children was 71.9%, while the corresponding rate in the permanent teeth of 12-year-old Chinese children was 38.5%.^17^

Caries is considered a dietary–microbial disease,^28^ commonly caused by fermentation of simple carbohydrates, such as sucrose, by oral micro-organisms, especially streptococci and lactobacilli. *Streptococcus mutans* is considered the main cariogenic bacterium; it utilises carbohydrates in food as the substrate to produce acid through glycolysis in the oral environment. Most of all, high sugar consumption contributes to the ecological imbalance of the oral microbiota, which has been associated with the increased risk of caries among healthy children with normal salivary secretion.^20^ Because of the irreversibility of caries, prevention is clinically more important than treatment.

Recently, the use of natural plant products to treat or prevent oral diseases has become a popular focus of research,^10,12^ such as grape seed extract,^6^ green tea,^19^ cranberry proanthocyanidins^16^ and propolis.^1^

Lemon essential oil (LEO), a mixture of terpenes and oxygenated derivatives,^5^ has been reported to have antibacterial and antioxidant potential.^2,13^ Our previous studies have shown that LEO could inhibit *S. mutans* acid production without affecting bacterial growth and the oral microbiological environment.^29^ In addition, LEO can inhibit sucrose-dependent adherence and the expression of related genes (gtfB and gtfC).^12^ Gas chromatography-mass spectrometry analysis has shown that this essential oil consists of a mixture of terpenes (78.9%), alcohols, acids, aldehydes, and ester compounds. Chief among the terpenes is limonene (48%), followed by β-terpinene.^21^ However, it remains unclear whether LEO affects the other main cariogenic virulence factors. Based on a previous study,^21^ we optimised LEO into lemon essential oil microemulsion (LEOM) to improve the low solubility and volatility of LEO in water. This study aimed to examine the effect of LEOM on acid production and the activity of lactate dehydrogenase (LDH) under different glucose concentrations, representing a cariogenic factor, and relative levels of the ldh gene expression of *S. mutans* in the glycolytic metabolic pathway. In addition, this study aimed to identify the associated mechanism and efficacy of LEOM, as well as to provide a theoretical basis for the development of natural anticaries products applicable in different oral-sugar environments, e.g. after a meal.

## Materials and Methods

### Bacterial Strains and Culture Conditions

*S. mutans* UA159 (serotype c) was inoculated into the liquid medium trypticase peptone yeast broth (TPY) and incubated for 18 h in an anaerobic incubator (80% N_2_ and 20% CO_2_) at 37°C. The bacterial suspension was prepared with 1×10^8^ cfu/ml and then used in the following assay.

### Extraction of LEO and Preparation of Microemulsion

The LEO used in this study was obtained from the peels of the same batch of lemons, grown in the Sichuan province, China, and picked during the intermediate maturation stage characterised by greenish-yellow coloration. As in the method used in previous studies,^26^ LEO was extracted using a hydrodistillation technique and prepared as a 9-mg/ml microemulsion, according to the ratio of 3:5:2:10 of lemon essential oil to Tween-80-H_2_O to polyethylene glycol to medium. The microemulsion placed in a water bath at 45°C for 1 h, followed by an ultrasonic water bath at 20 kHz and 100 W for 1 h with an ultrasonic crusher (Sonics; Newton, CT, USA). The emulsion was kept in a refrigerator at 4°C until use.^24^ The constituents of LEO were analysed with gas chromatography coupled with mass spectrometry (GC–MS, Varian450 [Agilent; Santa Clara, CA, USA], with the capillary column VF-1701MS. The GC–MS results showed that the CLO contains limonene (48.5%), b-pinene (17.0%), 4-carene (8.5%) and auraptene (6.85%).^18^

### Growth Inhibition Experiment

The microdilution technique was used to obtain the minimum inhibitory concentration (MIC) of LEO against *S. mutans*.^14^ A LEO sample of 90 μl was added to 96-well plates containing TPY medium using the two-fold serial dilution method. The final concentrations were 0.563, 1.125, 2.25, 4.5 mg/ml. TYP liquid medium without LEO was used as the vehicle control group. Ten microliters of bacterial suspension diluted with TYP liquid medium at a ratio of 1:10 (v/v) were seeded into 96-well plates in triplicate for each condition. At 37°C, the plate was incubated under anaerobic conditions for 1 day, and the absorbance of the samples was determined at 540 nm with a spectrophotometer (Eppendorf; Hamburg, Germany). The MIC was defined as the minimal LEOM concentration to inhibit bacterial growth with less than 0.01 variation in absorbance, using sterile medium as a reference.^22^

To simulate different oral glucose environments, TYP medium samples containing glucose at concentrations of 0% (no glucose), 0.2% (low glucose), 1% (medium glucose), and 5% (high glucose) were prepared, and LEOM was diluted to four concentrations of 1/8 MIC, 1/4 MIC, 1/2 MIC, and MIC with TPY medium. The bacterial liquid was added to the above-mentioned medium samples with different concentrations of glucose, cultured anaerobically at 37°C for 24 h. The minimum LEOM concentration without bacterial precipitation was considered the MIC of *S. mutans* under each particular glucose concentration.

### Acidogenicity Assay

The bacterial suspension was added to the 4-ml groups at a ratio of 1:100, and the initial pH value of each group was measured using a pH meter (Yue Ping; Yueping, China). Each group was cultured in an anaerobic incubator at 37°C for 24 h, and the supernatant was collected after the sample was centrifuged at 3000 rpm for 5 min. The final pH value of each group was determined after centrifugation. The effect of LEOM on the acid production capacity of *S. mutans* was evaluated based on the difference between initial pH and final pH (ΔpH = pH_initial_ – pH_final_).

### Activity of Lactate Dehydrogenase Assay

The activity of LDH in each group was determined by using the reductive coenzyme I oxidation method with a lactate dehydrogenase activity detection kit (A202-2, Jiancheng Institute of Biological Engineering; Nanjing, China), according to the manufacturer’s instructions. According to the change in the A value, the LDH activity of each group was calculated based on the catalytic activity of bacteria per mg, as follows: LDH activity (U/mg) = (A_test tube_ – A_counter-care tube_) × 0.2/ (A_sta__ndard tube_ – A_counter-care tube_) × protein concentration.

### Quantitative Real-time Polymerase Chain Reaction (RT-PCR)

After 24-h anaerobic culture, bacterial suspension was added to every tube at a ratio of 1:10 (v/v), and inoculated under anaerobic conditions at 37°C for 18 h. Subsequently, the bacteria were collected for RNA extraction using a bacterial total RNA kit (E.Z.N.A. Bacterial RNA Kit, OMEGA; Norcross, GA, USA). Reverse transcription was performed using a First-Strand cDNA Synthesis Kit with random primers (Takara; Osaka, Japan). In addition, 16SrRNA was chosen as an internal reference, and sequences of ldh primers for RT-PCR were designed by Primer5, according to the GenBank sequence of *S. mutans* UA159 (Table 2).^11^ RT-PCR amplification was performed using a thermal cycler (ABI, Thermo Fisher; Waltham, MA, USA). PCR conditions were as follows: an initial denaturation at 95°C for 2 min, followed by 40 cycles of denaturation at 95°C for 10 s, annealing at 55°C for 15 s, and finally ending with extension at 72°C for 30 s. To evaluate amplification specificity, a melting curve analysis was performed at the end of each PCR run. The 2−ΔΔCT method was applied to calculate the threshold cycle values obtained from the melting curve.

### Statistical Analysis

For error reduction, all experiments were performed in triplicate and reproduced three separate times. The results of the experiment were recorded as mean values ± SD. All data were analysed using GraphPad Prism 5.0 (GraphPad Software; San Diego, CA, USA). Intergroup differences were estimated using one-way ANOVA. The least-significant difference test was applied to compare means among the groups. p-values < 0.05 were considered statistically significant.

## Results

The MICs of LEOM against *S. mutans* at 5% and 0% glucose concentrations were 1.125 mg/ml, while MIC at 0.2% and 1% glucose concentrations was 2.25 mg/ml. Accordingly, the following experiments were grouped (Table 1). The 1/2 MIC, 1/4 MIC, and 1/8 MIC LEOM diluents were prepared in TPY medium containing 0%, 0.2%, 1%, and 5% glucose, respectively.

**Table 1:** The MIC of LEOM against *S. mutans* at different glucose concentrations

Glucose concentration	LEOM concentration (mg/ml)				
	Control	MIC	1/8MIC	1/4MIC	1/2MIC
0%	0	1.125	0.141	0.282	0.563
0.2%	0	2.5	0.282	0.563	1.125
1%	0	2.5	0.282	0.563	1.125
5%	0	1.125	0.141	0.282	0.563

MIC: minimum inhibitory concentration; LEOM: lemon essential oil microemulsion.

**Table 2:** Sequences of primers

Gene	Primer sequence (forward and reverse)	Reference
16S rRNA	5′-AGCGTTGTCCGGATTTATTG-3′ 5′-CTACGCATTTCACCGCTACA-3′	Li et al. 2016
ldh	5′-ACTTCACTTGATACTGCTCGTT-3 5′-AACACCAGCTACATTGGCATGA-3	Li et al. 2016

**Fig 1:**
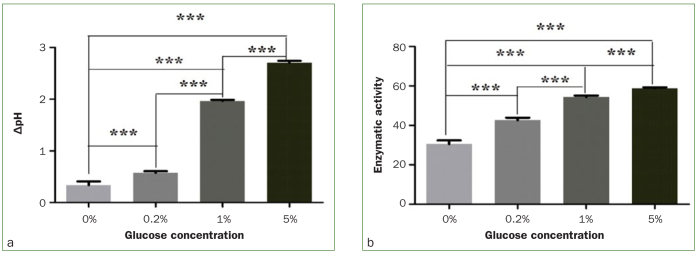
Effects of sugar concentration on acid production capacity and LDH enzyme activity of *S. mutans*. a. ΔpH under different sugar concentration; b. LDH activity of *S. mutans* at different glucose concentrations; *** indicates comparisons with 0% glucose concentration group, p < 0.001.

The results of acid production capacity and LDH enzyme activity analyses in *S. mutans* under different sugar concentrations are presented in Table 3 and Fig 1. Changes in pH and LDH activity in a glucose-free environment without LEOM were not statistically significant; thus, follow-up experiments did not involve a glucose-free environment. Among the different glucose concentration groups without LEOM, the glycolysis of *S. mutans* changed the pH value in the environment. The pH values decreased as glucose concentration increased. Pair-wise comparisons revealed differences in pH activity, depending on glucose concentration (p < 0.05). Changes to LDH activity showed similar trends.

**Table 3:** Acid production capacity and LDH enzyme activity of *S. mutans* under different glucose concentration

Glucose concentration	ΔpH	LDH enzyme activity
0%	0.34 ± 0.07	30.53 ± 1.85
0.2%	0.57 ± 0.03***	42.65 ± 1.22***
1%	1.97 ± 0.02***	54.37 ± 0.73***
5%	2.71 ± 0.04***	58.74 ± 0.55***

***p < 0.001.

**Fig 2:**
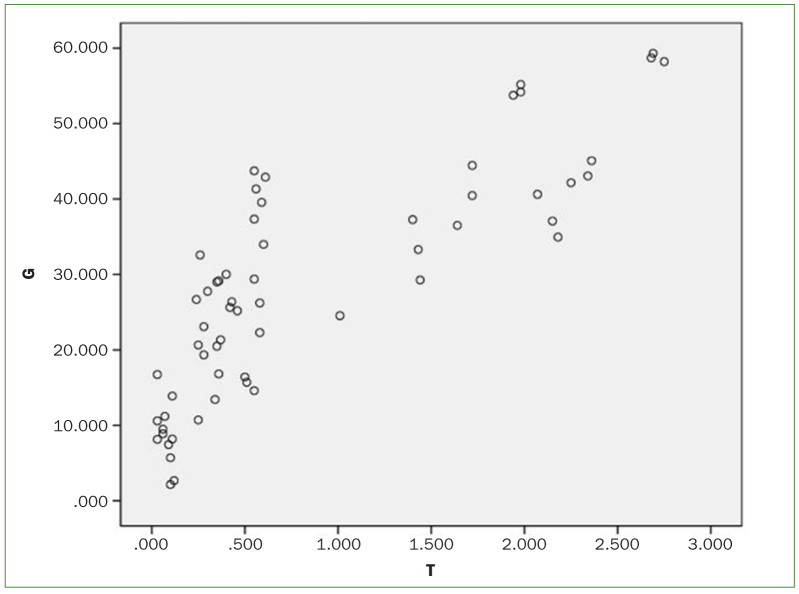
Correlation analysis of acid production levels and LDH activity of *S. mutans* at different glucose concentrations LDH, lactate dehydrogenase; G, enzyme activity; T, ΔpH.

As shown in Fig 2 and Table 4, at low glucose concentrations (0.2%), ΔpH was statistically significantly lower than in the control group in the presence of LEOM at only 1/2 MIC concentration (p < 0.05). At medium glucose concentration (1%), ΔpH was significantly lower than in the control group in the presence of LEOM at both 1/2 MIC and 1/4 MIC concentrations (p < 0.05). At high glucose concentration (5%), ΔpH was significantly lower than in the control group in the presence of LEOM at all concentrations (p < 0.05).

**Table 4:** Effects of LEOM on the acid production capacity of *S. mutans* at different glucose concentrations

ΔpH				
	Control	1/8 OIC	1/4 OIC	1/2 OIC
0.2%	0.57 ± 0.03	0.57 ± 0.02	0.52 ± 0.03	0.09 ± 0.03*
1%	1.97 ± 0.02	1.42 ± 0.02	0.63 ± 0.33*	0.07 ± 0.04*
5%	2.71 ± 0.04	2.32 ± 0.06*	2.13 ± 0.06*	0.26 ± 0.02*

*p < 0.05.

**Fig 3:**
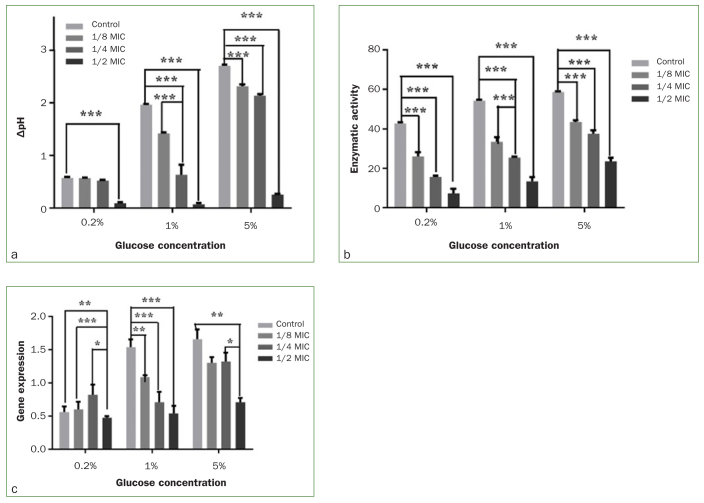
Effects of LEOM on acid production capacity (a), LDH enzyme activity (b), and ldh gene expression (c) of *S. mutans* at different sugar concentrations * p < 0.05; **p < 0.01; *** p < 0.001.

All concentrations of LEOM (1/2, 1/4, or 1/8 MIC) statistically significantly reduced the LDH activity of *S. mutans* under different glucose concentrations (p < 0.05) (Fig 2 and Table 5). Acid production was positively correlated with LDH activity of *S. mutans* at different glucose concentrations (r = 0.825, p < 0.05) (Fig 3).

**Table 5:** Effect of LEOM on the activity of *S. mutans* LDH at different glucose concentrations

	Control	1/8 MIC	1/4 MIC	1/2 MIC
0.2%	42.65 ± 1.22	25.96 ± 3.55*	15.56 ± 0.91*	7.11 ± 4.27*
1%	54.37 ± 0.73	33.28 ± 4.01*	25.36 ± 0.92*	13.37 ± 3.65*
5%	58.74 ± 0.55	43.42 ± 1.50*	37.55 ± 2.86*	23.47 ± 3.03*

MIC: minimum inhibitory concentration; *p < 0.05.

The virulent ldh gene expression levels in *S. mutans* are shown in Fig 2 and Table 6. The 1/2 MIC concentration of LEOM statistically significantly reduced the expression of ldh in the low (0.2%) and high glucose groups (5%) (p < 0.05) vs that observed in the control group. In the medium glucose group (1%), all three concentrations of LEOM statistically significantly reduced the expression of ldh (p < 0.05).

**Table 6:** Effect of LEOM on *S. mutans* ldh gene expression at different glucose concentrations

	Control	1/8 MIC	1/4 MIC	1/2 MIC
0.20%	1.43 ± 0.23	1.60 ± 0.20	1.16 ± 0.39	0.65 ± 0.20**
1%	1.54 ± 0.20	0.79 ± 0.31**	0.71 ± 0.27***	0.54 ± 0.20***
5%	1.42 ± 0.19	1.08 ± 0.17	1.32 ± 0.23	0.71 ± 0.11**

MIC: minimum inhibitory concentration; *p < 0.05, **p < 0.01, ***p < 0.001.

## Discussion

The oral microbiome plays an important role in the human microbial community and health.^7^ It is essential to inhibit the virulence factors associated with caries without disturbing the micro-ecological balance in the oral cavity. Based on previous findings, this study aimed to analyse the inhibitory effects of LEOM on the activity of cariogenic factors of *S. mutans* and associated mechanisms. First, this study found that acid production of *S. mutans* statistcally significantly increased with increasing glucose concentrations from 0%, 0.1%, 1%, to 5%. Next, we studied the influence of subminimum inhibitory concentration (sub-MIC) of LEOM on the acid production capacity of *S. mutans* at different glucose concentrations, revealing that all examined concentrations of LEOM inhibited the acid production capacity of *S. mutans* at different glucose concentrations. Moreover, the higher the glucose concentration, the more pronounced the LEOM inhibition effect of *S. mutans* acid production. These results indicated that LEO could down-regulate *S. mutans* acid production in a high-carbohydrate environment with high caries risk, such as the oral environment after a meal. Under high glucose conditions, *S. mutans* can synthesise large amounts of LDH, the key enzyme regulating lactic acid synthesis, while mainly utilising sugars to produce lactic acid. Meanwhile, under low glucose conditions, *S. mutans* experiences ‘sugar starvation’, whereby LDH activity is inhibited, pyruvate lyase is synthesised in large amounts, and sugars are mainly metabolised to generate ethanol, formic acid, and acetic acid. The pH values of formic acid and acetic acid are higher than those of lactic acid.^23^ This study has shown that LEOM can effectively inhibit the acid production of *S. mutans*, regardless of glucose concentration, and thus maintain the desired pH of the oral environment.

Lactic acid is the main cause of caries; it demineralises the tooth surface due to a decrease in the pH of dental plaque biofilms following bacterial metabolism of carbohydrates. In bacteria, LDH is an isoenzyme which catalyses the mutual conversion of lactic acid and pyruvate in the *S. mutans* glycolysis pathway. It is the dominant enzyme of the ‘lactic acid gate’ and the terminal enzyme of lactic acid synthesis in the Embden-Meyerhof pathway, which plays a key role in the bacterially-mediated cariogenic process. Therefore, LDH is a vital virulence factor.^9,24^ In a normal oral environment, the pH value of dental plaque is neutral or nearly neutral; the pH value decreases rapidly within 5–10 min after carbohydrate intake and slowly increases over approximately 40 min, to return to neutral levels. When the pH of the plaque drops below 5.5, the enamel is slightly demineralised; when the pH returns to normal, the enamel can remineralise. If these processes are balanced, the teeth can maintain a healthy state. In the present study, LDH activity was higher under glucose conditions than in the glucose-free environment, and it increased with an increase in glucose concentration. LDH activity in the three subinhibitory LEOM concentration groups was statistically significantly lower than that in the control group. These findings suggest that LEOM reduced the acidity of the environment and inhibited LDH activity. Furthermore, these two effects were positively correlated (r = 0.825).

To further explore the mechanism by which LEOM inhibits acid production in *S. mutans*, the effect of LEOM on lhd gene expression in *S. mutans* was examined by RT-PCR. In low (0.2%) and high glucose (5%) environments, the lhd gene expression was downregulated by LEOM at 1/2 MIC concentration. In addition, lhd gene expression was downregulated by LEOM at all three subinhibitory concentrations in the medium-glucose (1%) environment. LEOM inhibited the expression of the lhd gene of *S. mutans* in the presence of glucose; this inhibition appeared more effective at medium glucose concentrations. In addition, LEOM reduced the acid production of *S. mutans* by inhibiting ldh gene expression. However, the effects of LEOM on ldh gene expression and LDH activity were inconsistent in previous studies; some scholars believe that the reason for this phenomenon is that gene transcription includes mRNA transcription and protein translation.^26^ Protein translation requires complex processing and modification links, and the correlation coefficient between protein and mRNA levels is only 0.4–0.5. The activity of a gene translation enzyme associated with high transcription may not necessarily be high, and it is not necessarily positively correlated with the acid-producing capacity or cariogenicity of *S. mutans*.

LEOM, as a natural product, is mainly composed of limonene, its isomers, and other active ingredients. Preliminary evidence has shown that low concentrations of LEO can inhibit the growth, proliferation, and attachment of bacteria, but do not eliminate bacteria, which may help maintain the microecological balance of the oral environment. In addition, there was no cytotoxic reaction at effective bacteriostatic and antiviral concentrations, and the acute toxicity LD50 test findings have shown that the extract was non-toxic.^8^ Our research group proved that the single component had weaker antibacterial and anti-bacterial adhesion ability than the mixture in preliminary experiments.^3^ Based on Chinese medicine theory, the superposition of multiple components in each target and the synergistic action of different components between each target are one of the essential mechanisms of pharmaceutical effect. However, the targets and signal pathways of LEO on cariogenic dental plaque factors deserve further study. The bacteriostatic effect of the LEOM used in this experiment was stronger than those of previously examined LEO aqueous solutions. This result showed LEO microemulsion to be a better form, which could improve its low water solubility and volatilisation, thus enhancing its antibacterial effect. Finally, a limitation of this study must be mentioned: all results are based on *S. mutans* in vitro, which cannot fully mimic the oral environment. Further studies should try to use more complex model to explore anti-caries products effects and mechanisms.

## Conclusion

LEOM inhibited the expression of the ldh gene in *S. mutans* in different glucose environments, reduced LDH activity, and inhibited acid production by bacteria at subinhibitory concentrations without affecting the growth of bacteria. The present findings suggest that LEOM may become a new option among caries prevention materials. Considering the multifactorial nature of caries and the ecology of caries pathogenesis, more antibacterial experimental evidence simulating the oral environment should be gathered.
